# Danggui-Jakyak-San ameliorates memory impairment and increase neurogenesis induced by transient forebrain ischemia in mice

**DOI:** 10.1186/1472-6882-13-324

**Published:** 2013-11-22

**Authors:** Mi Deok Song, Dong Hyun Kim, Jong Min Kim, Hyung Eun Lee, Se Jin Park, Jong Hoon Ryu, Jae Hwan Lew

**Affiliations:** 1 Graduate School of East–west Medical Science, Kyung Hee University, Yongin 446-701, Republic of Korea; 2 Department of Life and Nanopharmaceutical Sciences, Kyung Hee University, Seoul 130-701, Republic of Korea; 3 Department of Oriental Pharmaceutical Science, College of Pharmacy, Kyung Hee University, Seoul 130-701, Republic of Korea

**Keywords:** Danggui-Jakyak-San, Transient forebrain ischemia, Neurogenesis, GSK-3β, β-catenin, Hippocampus

## Abstract

**Background:**

Danggui-Jakyak-San (DJS), a traditional herbal prescription, has been used to treat insufficient blood supplies. Recently, regenerative medication for the treatment of cerebral ischemia has drawn the attention of many researchers.

**Methods:**

In this study, we examined whether DJS exerts a neuronal regenerative effect in the hippocampus of a transient forebrain ischemia mice model. Transient forebrain ischemia was induced by bilateral common carotid artery occlusion (BCCAO). Animals were divided into three groups (sham, BCCAO + vehicle, and BCCAO + DJS). To test the effect of DJS on learning and memory, Morris water maze or passive avoidance test was conducted. To test neuroprotective and neurogenic effect, immunohistochemistry and Western blot analysis were used. Statistical significance was analyzed with Student *t*-test, one-way or two-way analysis of variance.

**Results:**

We found that the administration of DJS ameliorated ischemia-induced spatial memory impairment in the Morris water maze task. Moreover, Akt/glycogen synthase kinase-3β (GSK3β)/β-catenin signaling was increased by DJS, which would be one possible mechanism of DJS for neurogenesis in the hippocampal dentate gyrus region.

**Conclusions:**

These results suggest that DJS is a possible candidate for the treatment of ischemia-induced neuronal degeneration.

## Background

Neurogenesis in subgranular zone of hippocampus and the subventricular zone is affected by various physical, pharmacological, and pathological conditions, specifically including ischemic stroke and seizure [1,2]. Because of the regional correlation, neurogenesis in the hippocampal dentate gyrus (DG) has been studied in various disorders that cause memory impairment. As a result of these efforts, many anti-depressants or mood stabilizers have already been found to improve the neurological outcomes through an increase in neurogenesis in ischemic stroke models, including the enhancement of learning and memory, mood stability, and motor function [3].

Dangui-Jakyak-San (Danggui-Shaoyao-San in Chinese; Toki-Shakuyaku-San in Japanese; DJS) is an herbal prescription that consists of Paeoniae Radix, Atractylodis Rhizoma, Alismatis Rhizoma, Hoelen, Cnidii Rhizoma, and Angelicae Gigantis Radix. DJS has been widely used in the treatment of various types of diseases [4,5]. Previous clinical reports demonstrated that DJS has effects on the mild cognitive impairment [6] and functional deficits in post-stroke patients [7]. DJS also showed improvements of spatial cognition impairment [8,9] and dysfunction of central cholinergic nervous system [10] in animal models. Moreover, DJS has neuroprotective effects in vitro system [11,12]. However, functional mechanisms of the effect of DJS on various neurological deficits are still not clarified.

In a preliminary study, we found that long-term administration of DJS enhances learning and memory in normal naïve mice and increases neurogenesis in the hippocampus. Based on these findings, we speculated that DJS may improve the functional outcomes in an ischemic model through neurogenesis-induced neuronal regeneration. In a pilot study, the most effective dose of DJS was 100 mg/kg, and we adopted this dose for further experiments. We observed that delayed long-term treatment with DJS ameliorates the memory impairment induced by transient forebrain ischemia and that this effect may be due to the enhancing effects of DJS on neurogenesis.

## Methods

### Animals

Male C57BL/6 mice (22–26 g, 7 weeks) were purchased from the Orient Co. Ltd, a branch of Charles River Laboratories (Seoul, Korea), and kept in the University Animal Care Unit for 1 week prior to the experiments. The animals were housed 5 per cage, allowed access to water and food ad libitum; the environment was maintained at a constant temperature (23 ± 1°C) and humidity (60 ± 10%) under a 12-h light/dark cycle (the lights were on from 07:30 to 19:30 h). Thirty mice were divided equally into three groups (sham, n = 10; bilateral common carotid artery occluded ischemia + vehicle, n = 10; bilateral common carotid artery occluded ischemia + DJS, n = 10) for behavioral tests. The treatment and maintenance of the animals were carried out in accordance with the Animal Care and Use Guidelines of Kyung Hee University, Korea. All of the mouse experiments were performed according to the protocols approved by the Institutional Animal Care and Use Committee of Kyung Hee University (approved protocol numbers: KHP-2010-6-4; KHP-2010-8-11).

## Materials

5-Bromo-2′-deoxyuridine (BrdU) and crezyl violet were purchased from Sigma Chemical Co. (St. Louis, MO). Zoletil 50® was obtained from the Virbac laboratory (06516 Carros, France). The Complete Protease Inhibitor Cocktail and PhosSTOP Phosphatase Inhibitor Cocktail were purchased from Roche (Palo Alto, CA). The rat monoclonal anti-BrdU (ab6326) and rabbit polyclonal anti-Ki-67 (ab15580) antibodies were purchased from Abcam (Cambridge, UK). The goat polyclonal anti-doublecortin (DCX, sc-8066), rabbit polyclonal anti-Akt (sc-8312), goat polyclonal anti-tubulin (sc-9935), and goat polyclonal anti-GSK3β (sc-8257) antibodies were purchased from Santa Cruz Biotech (Santa Cruz, CA). The mouse monoclonal anti-NeuN (MAB377) and rabbit monoclonal anti-GFAP (AB5804) antibodies were purchased from Millipore (Temecula, CA). The rabbit polyclonal anti-pAkt (#9271), anti-pGSK3β (#9323), and anti-β-catenin (#9582) antibodies were purchased from Cell Signaling Technology Inc. (Danvers, MA). The avidin-biotin-peroxidase complex (ABC) kit was purchased from Vector Laboratories, Inc. (Burlingame, CA). All of the other materials were of the highest grade available and were obtained from normal commercial sources.

### Preparation of herbal extracts

The herbal materials [Paeoniae Radix (*Paeonia lactiflora* Pall, Paeoniaceae), Atractylodis Rhizoma (*Atractylodes japonica* Koidzmi, Compositae), Alismatis Rhizoma (*Alisma orientalis* Juzep, Alismataceae), Hoelen (*Poria cocos* Wolf, Polyporaceae), Cnidii Rhizoma (*Cnidium officinale* Makino, Umbelliferae), and Angelicae Gigantis Radix (*Angelica gigas* Nakai, Umbelliferae)] were purchased from the Kyungdong oriental drug store (Seoul, Korea) and identified by emeritus professor Chang Soo Yook, College of Pharmacy, Kyung Hee University. The DJS was prepared by boiling Paeoniae Radix (4 g), Atractylodis Rhizoma (4 g), Alismatis Rhizoma (4 g), Hoelen (4 g), Cnidii Rhizoma (3 g), and Angelicae Gigantis Radix (3 g) in 10 volumes of water. The aqueous solutions obtained were filtered, concentrated in a water bath under vacuum, frozen, lyophilized (Eyela, model FDU-2000, Japan), and stored at −20°C until required (yield; 12.3%). Paeoniflorin in Paeoniae Radix, ferulic acid in Cnidii Rhizoma, and decursin in Angelicae Gigantis Radix were used to ensure preparation consistencies. Paeoniflorin, ferulic acid, and decursin were present in DJS at 1.76%, 0.09%, and 0.38%, respectively.

### Passive avoidance test

To investigate memory enhancing effects of DJS, the acquisition and retention of passive avoidance behavior were conducted. The animals underwent two separated trials, an initial acquisition trial and a retention trial 24 h later. For the acquisition trial, a mouse was initially placed in the light compartment, and the door between the two compartments was opened 10 s later. When the mouse entered the dark compartment, the guillotine door automatically closed and an electrical foot shock (0.25 mA, 3 s) was delivered through the floor. For the retention trial, the mouse was again placed in the light compartment and the time required to enter the dark compartment was recorded. Mice were treated with DJS for 14 days and introduced into the acquisition trial 1 h after the last administration.

### Surgery and drug administration

C57BL/6 mice were anesthetized with 2.0% isoflurane and 70% nitrous oxide in oxygen and subjected to transient forebrain ischemia. The transient forebrain ischemia was induced by bilateral common carotid artery occlusion (BCCAO) with aneurysm clips for 20 min, and the circulation was restored by removing the clips. Mice that received the same surgical operation without carotid artery clipping served as sham-operated controls. During the surgical procedure, the rectal temperature was maintained at 37 ± 0.5°C with a heating pad (Biomed S.L., Spain). The regional cerebral blood flow (rCBF) was monitored using laser Doppler flowmetry (Perimed, PF5010, JarFalla, Sweden). The mice that showed between 80% and 95% reduction of rCBF were used in the study. After reperfusion, the animals were placed in a warm incubator (32–33°C) and returned to their home cages. DJS, which was dissolved in 10% Tween 80 solution, was administered from 7 days to 35 days after BCCAO (100 mg/kg, p.o., once daily) in the BCCAO + DJS group. BCCAO + vehicle group was administered with the same schedule with vehicle (10% Tween 80 solution) instead of DJS.

**Figure 1:**
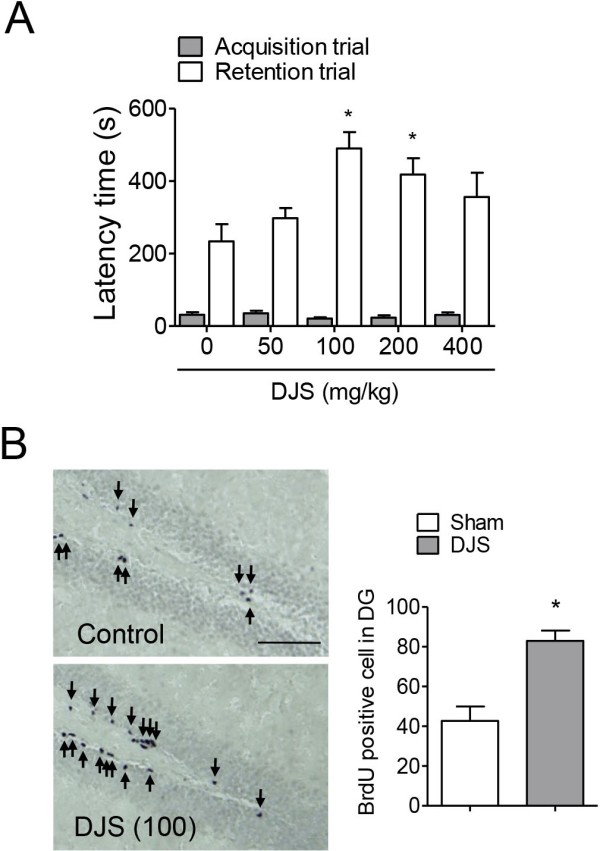
**The memory enhancing effect of Dangui-Jakyak-San (DJS). (A)** To test memory enhancing effect of DJS, passive avoidance test was conducted. DJS (50, 100, 200, and 400 mg/kg/day) was administered for 14 days. Passive avoidance task was conducted 1 h after the last treatment. Data are presented as mean ± S.E.M (n = 10/group). *P < 0.05, compared with control group (one-way ANOVA followed by Student Newman-Keuls’s *post-hoc* test). **(B)** In a separate experiment, BrdU staining was conducted to test effect of DJS on neurogenesis. Mice were treated with DJS (100 mg/kg/day), and sacrificed 1 h after the last administration. BrdU was administered at 13^th^ day (50 mg/kg, i.p., 3 times by 2 h interval). Arrows indicate immunopositive cells. Data are presented as mean ± S.E.M (n = 6/group). *P < 0.05, compared with control group (Student *t*-test). Bar = 50 μm.

### Morris water maze task

The Morris water maze task was conducted from 30 to 35 days after BCCAO and started 1 h after the administration of DJS. The first experimental day was dedicated to swim training for 60 s in the absence of the platform. During the four subsequent days, the mice were given one trial per session per day with the platform in place to avoid the ceiling effects induced by four trials per session. The starting point was changed for each training trial day. When a mouse located the platform, it was permitted to remain on it for 10 s. If the mouse did not locate the platform within 120 s, it was placed on the platform for 10 s. The animal was taken to its home cage and was allowed to dry off under an infrared lamp after each trial. During each trial, the time taken to find the hidden platform (escape latency) was recorded using a video camera-based Ethovision System (Nodulus, Wageningen, The Netherlands). One day after the last training trial, the mice were subjected to a probe trial session in which the platform was removed from the pool, allowing the mice to swim for 120 s to search for it. A record was kept of the swimming time in the pool quadrant where the platform had previously been placed.

### BrdU treatment and tissue preparation

Normal naive mice without introduction into BCCAO were treated with DJS for 14 days, and the mice were anesthetized with Zoletil 50® (10 mg/kg, i.m.) and then perfused transcardially with a 100 mM phosphate buffer (pH 7.4) followed by ice-cold 4% paraformaldehyde. The brains of the mice were removed and postfixed in a phosphate buffer (50 mM, pH 7.4) containing 4% paraformaldehyde overnight, then immersed in a 30% sucrose solution (in 50 mM phosphate-buffered saline, PBS), and stored at 4°C until sectioned. BrdU (50 mg/kg, i.p., 3 times by 2 h interval) was administered at 13^th^ day. In the ischemia experiment, mice were injected with BrdU (50 mg/kg, i.p., 3 times by 2 h interval) 7 days after BCCAO. Six mice in each group were anesthetized with Zoletil 50® (10 mg/kg, i.m.) 35 days after BCCAO, immediately after the probe trial. The perfusion and fixation were conducted as described above. The frozen brains were coronally sectioned on a cryostat at 30 μm and then stored in a storage solution (30% ethylene glycol, 30% glycerin, and 20 mM phosphate buffer) at 4°C. Hippocampal sections were collected based on the mouse brain atlas [13].

### Immunohistochemistry

The free-floating sections were incubated for 24 h in PBS (4°C) containing a rat anti-BrdU antibody (1:500), a rabbit anti-Ki67 antibody (1:1000) or a goat anti-DCX antibody (1:500), 0.3% Triton X-100, 0.5 mg/ml bovine serum albumin, and 1.5% goat or rabbit serum from the ABC kit. The sections were then incubated for 90 min with a biotinylated secondary antibody (1:200), treated with the ABC solution (1:100) for 1 h at room temperature, and reacted with 0.02% 3, 3′-diaminobenzidine tetrahydrochloride (DAB) and 0.01% H_2_O_2_ for approximately 3 min. After each incubation step, the sections were washed three times with PBS for 5 min. Finally, the sections were mounted on gelatin-coated slides, dehydrated in an ascending alcohol series, and cleared in xylene.

For multiple staining, free-floating sections were rinsed extensively with PBS (50 mM, pH 7.4) and then incubated for 30 min in HCl (2 N) at 37°C to denature DNA. Serial procedures were then conducted as described above. The BrdU-labeled cells were visualized with 0.02% DAB, 0.01% H_2_O_2_, and 0.2% nickel ammonium sulfate in 50 mM PBS for approximately 3 min. The free-floating sections were then incubated for 24 h in PBS (4°C) containing a rabbit anti-glial fibrillary acidic protein (GFAP) antibody (1:1000), a mouse anti-neuronal nuclei (NeuN) antibody (1:1000), or a goat anti-DCX antibody (1:500), as well as 0.3% Triton X-100 and 2% donkey serum. The sections were then incubated for 90 min with a FITC- or Cy3-conjugated secondary antibody (1:200). After each incubation step, the sections were washed three times with PBS for 5 min, finally, the sections were mounted on gelatin-coated slides, overlaid with VECTASHIELD mounting medium, and covered with a coverslip.

### Western blot analysis

For the preparation of Western blot samples, four mice were sacrificed immediately after the probe test of Morris water maze task, and their isolated hippocampal tissues were homogenized in an ice-cold Tris–HCl buffer (20 mM, pH 7.4) containing 0.32 M sucrose, 1 mM EDTA, 1 mM EGTA, 1 mM PMSF, a Complete Protease Inhibitor Cocktail (1 tablet/50 ml) and a PhosSTOP Phosphatase Inhibitor Cocktail (1 tablet/10 ml). Samples of the homogenates (20 μg of protein) were then subjected to SDS-PAGE (8%) under reducing conditions. The proteins were transferred to PVDF membranes in a transfer buffer [25 mM Tris–HCl buffer (pH 7.4) containing 192 mM glycine and 20% v/v methanol] and further separated at 400 mA for 2 h at 4°C. The Western blots were then incubated for 1 h with a blocking solution (5% skim milk), then with rabbit anti-pAkt, rabbit anti-pGSK3β, or rabbit anti-β-catenin (1:3000) antibody overnight at 4°C, washed ten times with Tween 20/Tris-buffered saline (TTBS), incubated with a 1:2000 dilution of horseradish peroxidase-conjugated secondary antibodies for 2 h at room temperature, washed ten times with TTBS, and finally developed by enhanced chemiluminescence (Amersham Life Science, Arlington Heights, IL). The blots were then stripped and incubated with a rabbit anti-Akt, rabbit anti-GSK3β or goat anti-tubulin antibody (1:5000). The membrane was analyzed with the bio-imaging program of the LAS-4000 mini (Fujifilm Lifescience USA, Stamford, CT).

### Nissl staining

After the sections were mounted onto gelatin-coated slides, they were stained with 0.5% cresyl violet, dehydrated through graded alcohols (70, 80, 90, and 100% × 2), placed in xylene, and covered with a coverslip after the addition of Histomount media.

### Quantification and statistical analysis

The number of cells in the hippocampal DG region was determined using a computerized image analysis system (Leica Microsystems AG, Wetzlar, Germany). The cells were counted in 6 sections by every 8 sections interval (total 48 sections) per animal by a person blind to the treatment group, and the average cell count per section was computed. The degree of damage in the hippocampal CA1 by the Nissl staining after ischemia was semi-quantitatively scored from 0 to 3 (0, normal; 1, < 30% of the neurons were irreversibly damaged; 2, 30–60% of the neurons were irreversibly damaged; 3, 60-100% of the neurons were irreversibly damaged) as described elsewhere [14]. The values are expressed as the means ± S.E.M. The results of histological, immunohistochemical, and Western blot analyses were analyzed using Student *t*-test or one-way analysis of variance (ANOVA) followed by Student Newman-Keuls’s *post-hoc* test for multiple comparisons. Two-way ANOVA was used to analyze the data of the path length in the training trials of the Morris water maze task, and when the results were significant, Bonferroni’s *post-hoc* test was used to compare the treatment groups. The statistical significance was set at *P* < 0.05.

## Results

### DJS enhanced learning and memory in the passive avoidance test and BrdU incooperation

DJS (100 or 200 mg/kg/day, p.o.)-administered normal mice showed significant increase in step-through latency during the retention trial of the passive avoidance task (*P* < 0.05, Figure 1A). However, there were no significant changes in step-through latency in the other treated groups (50 or 400 mg/kg). We adopted 100 mg/kg of DJS for further study. Moreover, DJS administration (100 mg/kg/day, p.o.) also increased the number of BrdU-positive cells in mouse hippocampal DG region (*P* < 0.05; Figure 1B).

**Figure 2:**
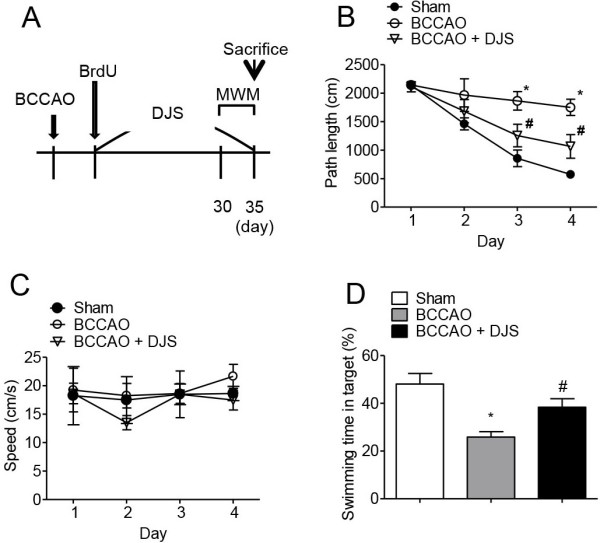
**The effect of Dangui-Jakyak-San (DJS) on ischemia-induced memory impairment.** To test the effect of DJS on ischemia-induced memory impairment, Morris water maze test was conducted after ischemic injury. Ischemic brain injury was induced by bilateral common carotid artery occlusion (BCCAO). **(A)** Experimental schedule. **(B-D)** The effect of DJS on the Morris water maze task. Path length **(B)** and swimming speed **(C)** were recorded during the training trials. Swimming time in target quadrant which platform was previously existed **(D)** was recorded in the probe trial. Data are presented as mean ± S.E.M (n = 10/group). *P < 0.05, compared with sham group. #*P* < 0.05, compared with BCCAO group (two-way ANOVA followed by Bonferroni’s *post-hoc* test for **B** and **C**, one-way ANOVA followed by Student Newman-Keuls’s *post-hoc* test for **D**).

### Delayed DJS treatment ameliorated memory impairment induced by transient forebrain ischemia

As shown in Figure 2B, DJS significantly shortened the path length (*P* < 0.05; Figure 2B) in the training trials without affecting the swimming speed (Figure 2C). In the probe trial, the swimming times within the target quadrant in the DJS-treated group were significantly higher than those in the vehicle-treated control group (*P* < 0.05, Figure 2D).

### Delayed DJS treatment increased neurogenesis

**Figure 3:**
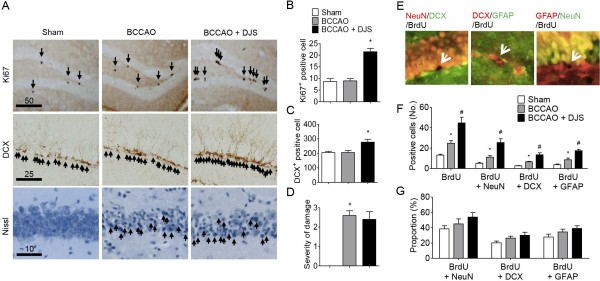
**The effect of Dangui-Jakyak-San (DJS) on ischemia-induced neurogenesis.** To test newborn cell survival and neurogenesis, Ki67 or DCX immunostaining was conducted. **(A)** Representative of microphotographs of Ki67- and DCX-, and nissl staining-positive cells in the hippocampal DG region (Bar = 10, 25 or 50 μm). Arrows indicate immunopositive cells. **(B)** The number of Ki67-immunopositive cells, **(C)** the number of DCX-immunopositive cells in the DG, and **(D)** severity of neuronal damage in the CA1 region were analyzed. **(E-G)** To test effect of DJS on ischemia-induced neural differentiation, double-immunostainings were conducted. **(E)** Representative of microphotographs of BrdU/NeuN-, BrdU/DCX-, and BrdU/GFAP-immunopositive cells in the hippocampal DG region. Bar = 30 μm. **(F)** BrDU-immunopositive cells, BrDU/NeuN-immunopositive cells, BrDU/DCX-immunopositive cells or BrDU/GFAP-immunopositive cells were counted. **(G)** Proportion of BrdU/NeuN-, BrdU/DCX-, and BrdU/GFAP-immunopositive cells to BrdU-immunopositive cells in the hippocampal DG region was analyzed. Data are presented as mean ± S.E.M (n = 6/group). *P < 0.05, compared with sham group. #*P* < 0.05, compared with BCCAO group (one-way ANOVA followed by Student Newman-Keuls’s *post-hoc* test).

In neural proliferation, cells positive for Ki67 were significantly increased in DJS-treated group compared with the other groups (*P* < 0.05, Figure 3A and B). Moreover, DCX-positive cells were significantly increased by DJS treatment (*P* < 0.05, Figure 3A and C). To test whether DJS has neuroprotective effect, we conducted Nissl staining of CA1 region which is well known as most vulnerable region in ischemic brain [15-17]. However, delayed DJS treatment did not protect neuronal injury induced by BCCAO (*P* > 0.05, Figure 3A and D), suggesting that effect of DJS is not due to its protective effect. In neuronal differentiation, the DJS-treated BCCAO group showed significantly higher levels of BrdU-positive cells (*P* < 0.05, Figure 3F), BrdU and NeuN-double-positive cells (*P* < 0.05, Figure 3E and F), BrdU and DCX-double-positive cells (*P* < 0.05, Figure 3E, and F], and BrdU and GFAP-double-positive cells (*P* < 0.05, Figure 3E and 3F) than those of the vehicle-treated BCCAO or sham groups. In addition, these neuronal differentiation markers were also slightly increased in the vehicle-treated BCCAO group. However, the propotions of each neural cell were not significantly different (Figure 3G).

### Delayed DJS treatment activated Akt/GSK3β/β-catenin signaling

**Figure 4:**
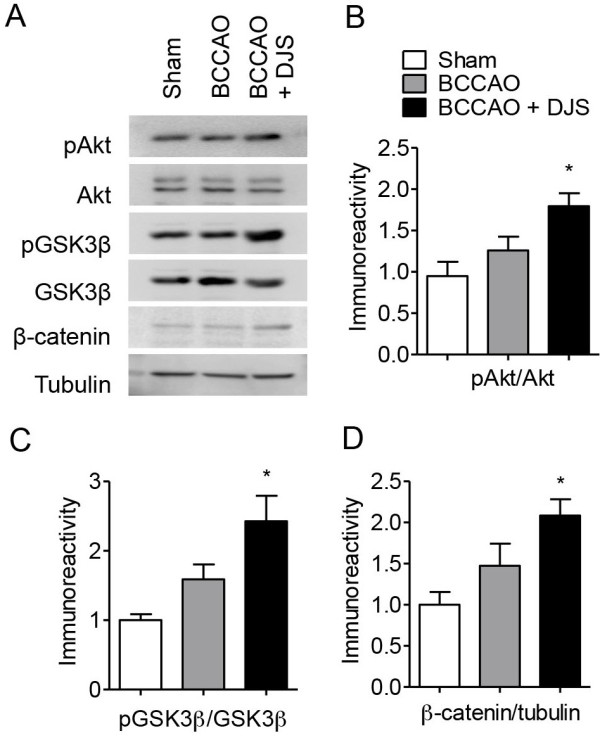
**The effect of Dangui-Jakyak-San (DJS) on ischemia-induced changes of GSK3β signaling.** Using Western blot assay, changes of GSK3β signaling were examined. **(A)** Representative photographs of Western blotting. Ratio of pAkt to Akt **(B)**, ratio of pGSK3β to GSK3β **(C)**, and ratio of β-catenin to tubulin **(D)** immunoreactivity in the hippocampal tissue were analyzed. Data are presented as mean ± S.E.M (n = 4). *P < 0.05, compared with sham group (one-way ANOVA followed by Student Newman-Keuls’s *post-hoc* test).

In the hippocampi of the vehicle-treated BCCAO group, the expression levels of pAkt, pGSK3β, and β-catenin were not significantly different from those of sham group. However, DJS treatment significantly increased these signaling molecules in the hippocampus (*P* < 0.05, Figure 4A-D).

## Discussion

There is still no successful treatment for massive neurodegenerative diseases such as ischemic stroke. Therefore, many researchers have been focusing on the regeneration of tissue or neurons using embryonic stem cells. However, methods utilizing these cells are still under preclinical study due to insufficient knowledge about their physiology. Recent findings in rodent models of stroke that suggest the replacement of hippocampal pyramidal cells by endogenous progenitors raise optimism for regenerative therapies [18]. Before using endogenous neuronal progenitors for the treatment of brain ischemia, several issues should be addressed. First, to achieve the replacement of lost neurons, neuronal progenitors should first change the pathway towards the ischemic lesion, where constitutive neurogenesis does not normally occur (non-neurogenic regions). The second issue is that few neurons actually mature in the lesion, which would account for the difficulty of functional recovery after ischemic injury. It has also been questioned whether such new neurons become functionally active. Recently, these issues were addressed through various experiments. Previous report has already suggested that treatment with antidepressant and constituents of ginseng increased ischemic stroke-induced neurogenesis and improved the functional outcome [3]. Moreover, blockade of neurogenesis in ischemic brain exacerbates functional deficit [19]. They suggest that the increase of neurogenesis give a chance to overcome loss of neurons in ischemic vulnerable region. Therefore, these findings suggest that the increase of endogenous neurogenesis may be good target for repairing neurodegenerative diseases.

DJS has shown neuroprotective effect in various in vivo and in vitro conditions [6-12]. However, functional mechanisms of the effect of DJS on various neurological deficits are still not clarified. Previous reports indicated that DJS regulates neurotransmitters level including acetylcholine and monoamines, and reduces oxidative damage in various brain disease model [8,11,20]. In this study, we found that delayed long-term administration of DJS improves ischemic damage-induced spatial memory impairment. In addition, DJS significantly increased transient forebrain ischemia-induced hippocampal neurogenesis and attenuated memory impairment. Moreover, because delayed administration with DJS failed to rescue ischemia-induced neuronal cell death, we suggest that DJS-induced increase of neurogenesis may be a possible mechanism for functional improvement. However, it was found that DJS did not affect neuronal differentiation. Further research should be required to clarify this issue. While DJS showed its memory enhancing effect at 14-day treatment in normal naïve mice, it showed its effect at 28-day but not at 14-day treatment in ischemic mice. The reasons of the differences are unclear, but we speculate that effective compounds, which are contained in DJS, are different in depends on conditions.

Recently, it has been suggested that GSK3 is a master regulator of neuronal progenitor homeostasis [21]. Changes in GSK3β activity occur after cerebral ischemia in regional, severity of injury, or model dependent manner: increase in cortical and striatal neurons after global ischemia [22]; decreases in the hippocampal CA1 but not CA3 region after global ischemia [23]. However, GSK3β activity has not been examined in the neurogenic regions including hippocampal DG and subventricular zone. In compatible with neurogenesis, DJS modulated ischemia-induced changes in Akt/GSK3β/β-catenin singaling. Appropriate regulation of β-catenin, which is a downstream molecule of GSK3β, is critical for the control of progenitior proliferation in various regions of the developing nervous system [24,25]. Therefore, GSK3β/β-catenin signaling is to be one possible mechanism for DJS-induced neurogenesis.

## Conclusions

Taken together, we propose that post-ischemic prolonged DJS administration ameliorates the memory impairment induced by transient forebrain ischemia via an increase of neurogenesis that is mediated by activation of hippocampal Akt/GSK3β/β-catenin signaling. However, treatment should be conducted carefully because the effect of DJS is not promise in higher dose.

## Abbreviations

DJS: Danggui-Jakyak-San; DG: Dentate gyrus; GSK3β: Glycogen synthase kinase-3β; BrdU: 5-Bromo-2′-deoxyuridine; DCX: Doublecortin; ABC: Avidin-biotin-peroxidase complex; BCCAO: Bilateral common carotid artery occlusion; rCBF: regional cerebral blood flow; PBS: Phosphate-buffered saline; TTBS: Tris-buffered saline; ANOVA: One-way analysis of variance; GFAP: Glial fibrillary acidic protein; NeuN: Neuronal nuclei.

## Competing interests

The authors declare no conflict of interests.

## Authors’ contributions

MDS designed and conducted all neurogenesis experiments. DHK and JMK conducted behavioural experiments. HEL and SJP conducted western blot and immunohistochemical experiments. JHR and JHL conceived of the study, participated in its design and coordination and helped to draft the manuscript. All authors read and approved the final manuscript.

## Pre-publication history

The pre-publication history for this paper can be accessed here:

http://www.biomedcentral.com/1472-6882/13/324/prepub
